# Structural basis for binding diversity of acetyltransferase p300 to the nucleosome

**DOI:** 10.1016/j.isci.2022.104563

**Published:** 2022-06-09

**Authors:** Suguru Hatazawa, Jiuyang Liu, Yoshimasa Takizawa, Mohamad Zandian, Lumi Negishi, Tatiana G. Kutateladze, Hitoshi Kurumizaka

**Affiliations:** 1 Laboratory of Chromatin Structure and Function, Institute for Quantitative Biosciences, The University of Tokyo, 1-1-1 Yayoi, Bunkyo-ku, Tokyo 113-0032, Japan; 2 Department of Biological Sciences, Graduate School of Science, The University of Tokyo, 1-1-1 Yayoi, Bunkyo-ku, Tokyo 113-0032, Japan; 3 Department of Pharmacology, University of Colorado School of Medicine, Aurora, CO 80045, USA

**Keywords:** Biological sciences, Biochemistry, Structural biology

## Abstract

p300 is a human acetyltransferase that associates with chromatin and mediates vital cellular processes. We now report the cryo-electron microscopy structures of the p300 catalytic core in complex with the nucleosome core particle (NCP). In the most resolved structure, the HAT domain and bromodomain of p300 contact nucleosomal DNA at superhelical locations 2 and 3, and the catalytic site of the HAT domain are positioned near the N-terminal tail of histone H4. Mutations of the p300-DNA interfacial residues of p300 substantially decrease binding to NCP. Three additional classes of p300-NCP complexes show different modes of the p300-NCP complex formation. Our data provide structural details critical to our understanding of the mechanism by which p300 acetylates multiple sites on the nucleosome.

## Highlights

• The cryo-EM structures of the p300-nucleosome complexes were determined
• The nucleosome binding residues in the HAT and bromodomain of p300 were identified
• p300 binds to the nucleosome in multiple binding modes

## Graphical abstract

**Figure:**
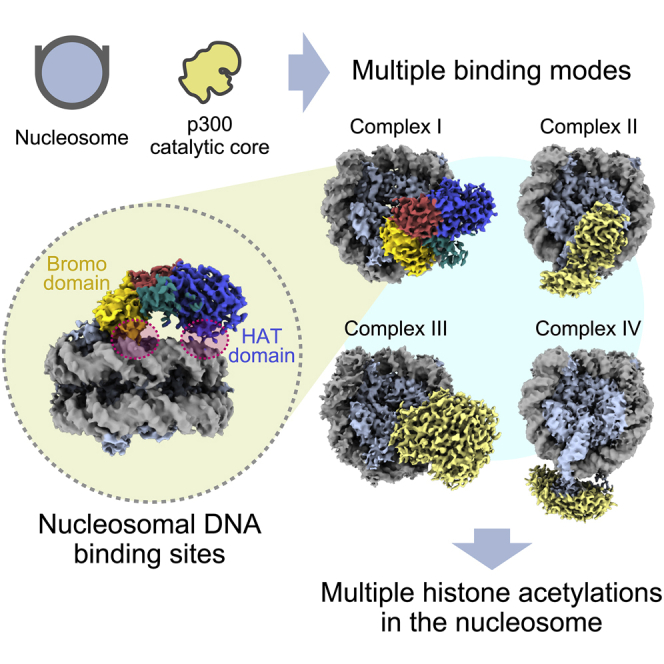

## Introduction

p300, a major human histone acetyltransferase (HAT), mediates vital biological processes and is linked to diseases, including cancer and neurodegeneration (Iyer et al., 2004; Lasko et al., 2017; Shin et al., 2021; Wang et al., 2013). p300 acetylates histones in the nucleosome (Ogryzko et al., 1996), the fundamental unit of chromatin, and alters chromatin structure and dynamics. However, the mechanism by which p300 associates with the nucleosome remains unknown.

In eukaryotic cells, histones H2A, H2B, H3, and H4, wrapped with genomic DNA, form the basic unit of chromatin, the nucleosome (Luger et al., 1997). Owing to the high stability of the nucleosome and its low DNA accessibility, many genomic functions, such as transcription, replication, recombination, and repair, in nucleosome-dense chromatin are generally suppressed (Lai and Pugh, 2017; Teves et al., 2014). One of the mechanisms to alleviate the nucleosome-driven suppression involves post-translational modifications (PTMs) of histones, particularly acetylations of lysine residues, which change the dynamics and structural properties of the nucleosome (Tessarz and Kouzarides, 2014; Zentner and Henikoff, 2013). Acetylation removes the positive charge from lysine residues, which are abundant in histones, thereby hindering the interactions with the negatively charged DNA and destabilizing the nucleosome. Acetylation is a major PTM that is generally associated with transcriptionally active chromatin, and it also recruits nucleosome-remodeling and nucleosome-modifying proteins and complexes for further activation of chromatin (Kouzarides, 2007; Musselman et al., 2012).

Among the major human acetyltransferases, p300 stands out since it acts on a broad range of substrates, including histones and non-histone proteins (Dancy and Cole, 2015; Gu and Roeder, 1997; Jin et al., 2011; Tang et al., 2013; Turnell et al., 2005; Weinert et al., 2018). The acetyltransferase function of p300 is required for diverse fundamental cellular processes, such as transcriptional activation, DNA damage repair, and stress response (An et al., 2004; Bose et al., 2017; Demarest et al., 2002; Hasan et al., 2001). Lysine residues in all four histones, H2A, H2B, H3, and H4, are efficiently acetylated by p300 (Ogryzko et al., 1996; Schiltz et al., 1999; Thompson et al., 2001). p300 acetylates K5, K8, K12, and K16 in the free histone H4 protein, but prefers H4K5 and H4K8 as substrates when H4 is incorporated within the nucleosome (Schiltz et al., 1999). Selective acetylations of H3K27 and H3K18 (Jin et al., 2011; Tang et al., 2013) by p300 require the cooperative action of its acetyllysine-binding bromodomain (BD) and the histone H3-binding ZZ domain (Zhang et al., 2018). In the context of the nucleosome, the predominant site for H2A acetylation by p300 is K5, whereas H2B is acetylated on K5, K12, K15, and K20 (Schiltz et al., 1999).

Despite the crucial role of p300 in chromatin acetylation, the mechanism by which p300 associates with the nucleosome has not been determined. To gain mechanistic insights into the basis for p300 binding to chromatin and the acetylation process, in the present study, we obtained the structure of the p300-nucleosome complex by cryo-electron microscopy (cryo-EM).

## Results and discussion

### Nucleosome binding by the catalytic core of p300

**Figure 1:**
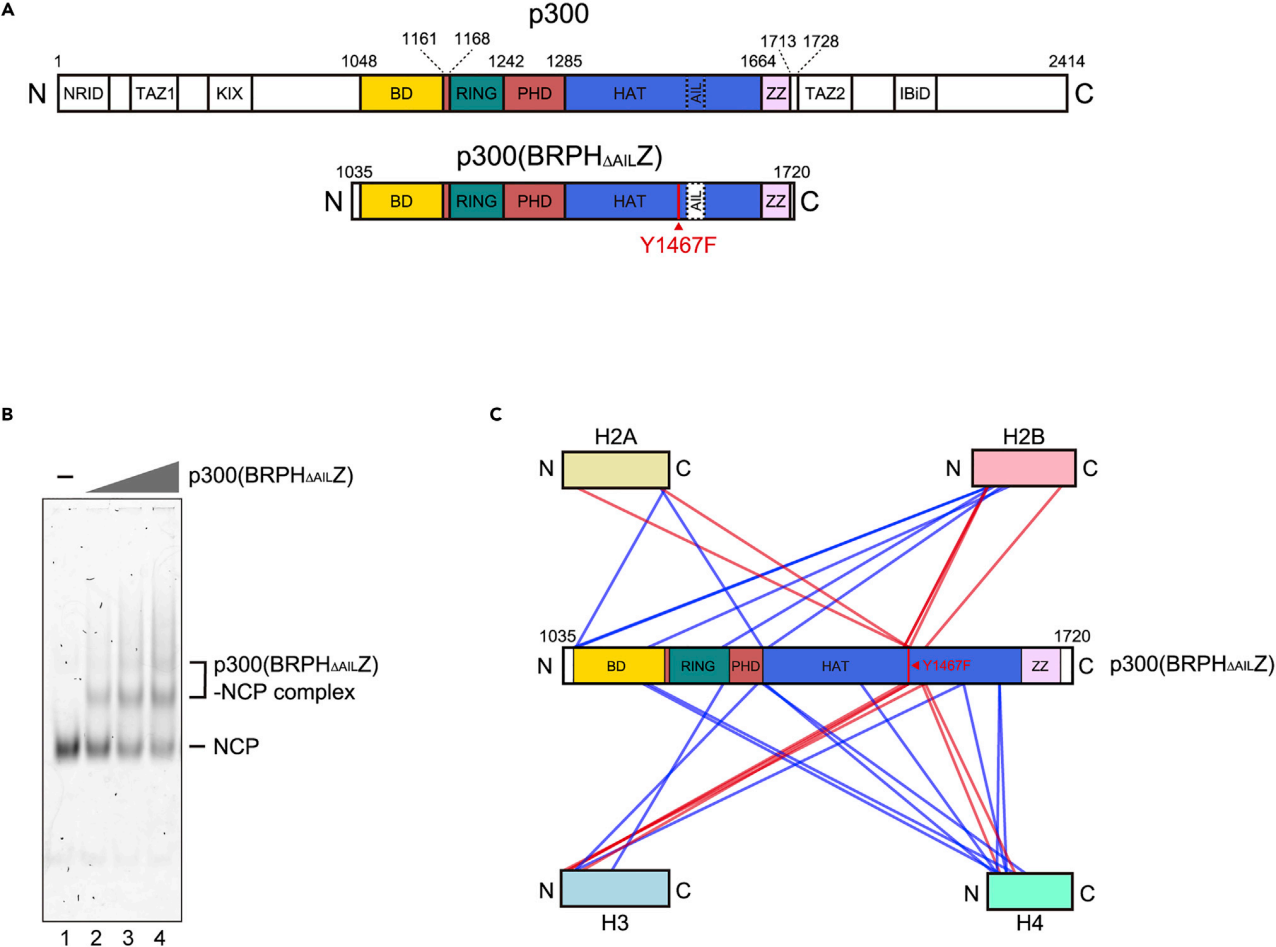
p300(BRPH_ΔAIL_Z) binds to the NCP (A) p300 and p300(BRPH_ΔAIL_Z) domain architectures. NRID: nuclear receptor interaction domain, TAZ1: transcriptional adaptor zinc-finger domain 1, KIX: kinase-inducible domain of CREB-interacting domain, BD: bromodomain, RING: really interesting new gene domain, PHD: plant homeodomain, HAT: histone acetyltransferase domain, AIL: autoinhibition loop, ZZ: ZZ-type zinc-finger domain, TAZ2: transcriptional adaptor zinc-finger domain 2, IBiD: IRF3-binding domain. p300(BRPH_ΔAIL_Z) has the Y1467F mutation and lacks the AIL. (B) Electrophoretic mobility shift assay of p300(BRPH_ΔAIL_Z) and the NCP. The p300(BRPH_ΔAIL_Z)-NCP complex formation was analyzed by non-denaturing 4% polyacrylamide gel electrophoresis with SYBR Gold staining. (C) Schematic representation of the results obtained by the crosslinking mass spectrometric analysis of the p300(BRPH_ΔAIL_Z)-NCP complexes. The inter-protein crosslinks between histones and p300(BRPH_ΔAIL_Z) are represented by lines. The crosslinks of the histone N-terminal regions with the p300(BRPH_ΔAIL_Z) residues near the HAT catalytic center are shown by red lines.

To study the p300-nucleosome interaction, we used the catalytic core of human p300 [p300(BRPHZ)], consisting of the bromodomain, the RING and PHD fingers, and the HAT and ZZ domains (Figure 1A). To prevent the dissociation of the p300-nucleosome complex, we generated the catalytically inactive Y1467F mutant and removed the autoinhibition loop, which is known to block the catalytic center of the p300 HAT domain in the p300(BRPH_ΔAIL_Z) construct (Figure 1A). The p300 Y1467F mutation was found as an amino acid substitution that abolishes the acetyltransferase activity of p300 (Liu et al., 2008). In contrast, the deletion of the autoinhibition loop reportedly enhances the acetyltransferase activity of p300, because of its augmented binding activity to target peptides (Thompson et al., 2004). The nucleosome core particle (NCP) was reconstituted with a 145 base-pair Widom 601 nucleosome positioning sequence (Figures S1A and S1B). Electrophoretic mobility shift assays (EMSAs) demonstrated that p300(BRPH_ΔAIL_Z) readily binds to the NCP, as we observed bands indicative of p300(BRPH_ΔAIL_Z)-NCP complex formation (Figure 1B). p300(BRPH_ΔAIL_Z) also binds to the naked 145 base-pair Widom 601 DNA (Figure S1C). Crosslinking mass spectrometry analyses revealed that all acetylation substrates of p300, including the N-terminal tails of H2A, H2B, H3, and H4 and the C-terminal tails of H2A and H2B, are located close to the catalytic center of the HAT domain in the p300(BRPH_ΔAIL_Z)-NCP complexes (Figure 1C). These results suggest that the p300(BRPH_ΔAIL_Z)-NCP complex can adopt multiple active forms for the acetylation of different histone tails.

### Cryo-EM structure of the p300(BRPH_ΔAIL_Z)-NCP complex (complex I)

**Figure 2:**
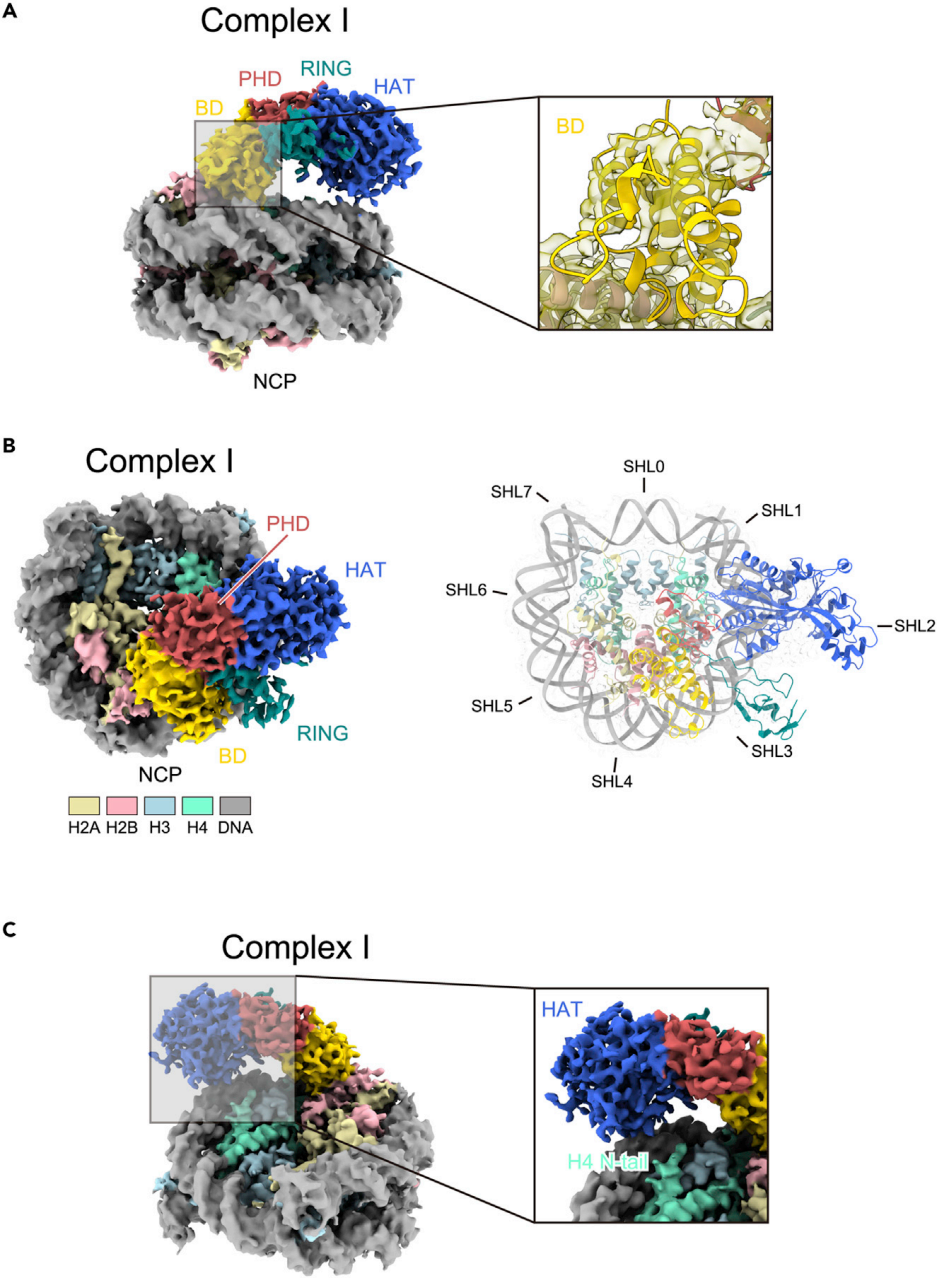
Cryo-EM structure of the p300(BRPH_ΔAIL_Z)-NCP complex I (A) Cryo-EM maps of the p300(BRPH_ΔAIL_Z)-NCP complex I. The overall structure and the enlarged bromodomain region are presented in the left and right panels, respectively. The bromodomain (BD), PHD domain, RING domain, and HAT domain of p300 are colored yellow, red, green, and blue, respectively. In the enlarged panel, the crystal structures of the NCP (PDB ID: 3LZ0) and the bromodomain of the p300 catalytic core (PDB ID: 5LKU) are fitted into the density map. (B) Top view of the p300(BRPH_ΔAIL_Z)-NCP complex I. In the right panel, the crystal structures of the NCP and p300 catalytic core are superimposed on the cryo-EM map. Superhelical locations (SHLs) of the NCP are shown. (C) Enlarged view of the H4 N-terminal tail region located near the p300 HAT domain.

The p300(BRPH_ΔAIL_Z)-NCP complex samples were fixed with glutaraldehyde (0.1%), fractionated by sucrose density gradient ultracentrifugation (Figures S2A and S2B), and used in cryo-EM data acquisition (Figures S2C, S2D, and S3) for the generation of the cryo-EM map of the p300(BRPH_ΔAIL_Z)-NCP complex (complex I) (Figure 2A, left, and Table 1). The p300(BRPH_ΔAIL_Z)-NCP complex structure was obtained by rigid body fitting, using the crystal structure of p300(BRPH_ΔAIL_) (PDB ID: 5LKU) (Kaczmarska et al., 2017) as a model (Figure S4). As shown in Figures 2A, 2B, and S4, four α-helices of the p300 bromodomain fitted well with the cryo-EM map in the p300(BRPH_ΔAIL_Z)-NCP structure.

**Table 1:** Cryo-EM data collection, refinement, and validation statistics for p300(BRPH_ΔAIL_Z)-NCP complexes

Sample	Complex I	Complex II	Complex III	Complex IV
	(EMD-32373, PDB 7W9V)	(EMD-32374)	(EMD-32375)	(EMD-32376)
**Data collection**				

Electron microscope	Krios G3i			
Camera	K3			
Magnification	81,000×			
Pixel size (Å/pix)	1.07			
Defocus range (μm)	−1.2 to −2.3			
Exposure time (second)	10			
Total dose (e/Å^2^)	1st dataset: 55.975, 2nd dataset: 56.149			
Movie frames (no.)	40			
Total micrographs (no.)	14,102			

**Reconstruction**				

Software	Relion 3.1			
Particles for 2D classification	8,811,235			
Particles for 3D classification	2,778,145			
Particles in the final map (no.)	25,884	145,147	25,208	227,652
Symmetry	C1	C1	C1	C1
Final resolution (Å)	3.95	3.38	3.95	3.29
FSC threshold	0.143	0.143	0.143	0.143
Map sharpening B factor (Å^2^)	−28.8	−30.0	−35.5	−35.9

**Model building**				

Software	Coot			

**Refinement**				

Software	Phenix			

**Model composition**				

Protein	1,302			
Nucleotide	290			

**Validation**				

MolProbity score	1.47			
Clash score	8.50			

**R.m.s. deviations**				

Bond lengths (Å)	0.006			
Bond angles (˚)	0.948			

**Ramachandran plot**				

Favored (%)	97.97			
Allowed (%)	2.03			
Outliers (%)	0			

The structure of the p300(BRPH_ΔAIL_Z)-NCP complex shows that the HAT domain and bromodomain of p300 contact the nucleosomal DNA at superhelical locations SHL2 and SHL3, respectively (Figures 2A and 2B). In contrast, the PHD and RING fingers do not directly contact the NCP, and the ZZ domain could not be visualized, probably due to its flexibility in the complex (Figure 2A). Although histone tails in general are not clearly visible in the p300(BRPH_ΔAIL_Z)-NCP complex I because of their flexible nature, the N-terminal region of H4 is appropriately positioned to bind within the catalytic center of the HAT domain (Figure 2C). Therefore, the complex I structure likely provides a structural basis for the H4 acetylation by p300.

### Mutational analyses of the nucleosomal DNA-binding sites of p300

**Figure 3:**
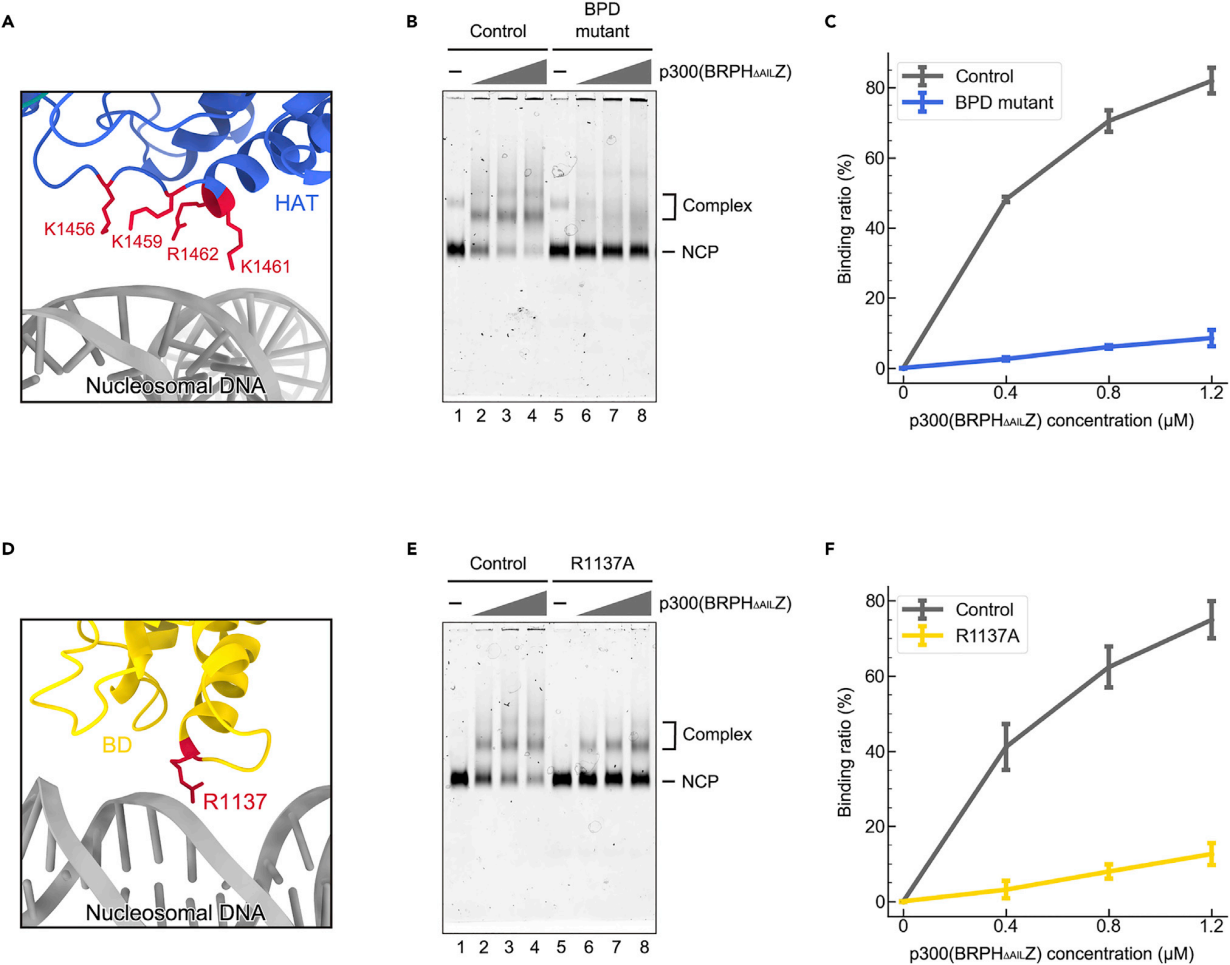
NCP-binding activities of the p300(BRPH_ΔAIL_Z) mutants (A) Close-up view of the interaction site between the p300(BRPH_ΔAIL_Z) HAT domain and the nucleosomal DNA. (B) Electrophoretic mobility shift assay of the p300(BRPH_ΔAIL_Z) BPD mutant (K1456A/K1459A/K1461A/R1462A) with the NCP. Complex formation was analyzed by non-denaturing 4% polyacrylamide gel electrophoresis with SYBR Gold staining. Lanes 1–4 are control experiments with p300(BRPH_ΔAIL_Z), and lanes 5–8 are experiments with the p300(BRPH_ΔAIL_Z) BPD mutant. (C) Quantitative results of the NCP-binding activity of the p300(BRPH_ΔAIL_Z) BPD mutant. Ratios of the NCP bound to p300(BRPH_ΔAIL_Z) were estimated from the band intensity of the remaining free NCP, and the average values of three independent experiments (shown in panel (B) and Figure S5A) are plotted against the p300(BRPH_ΔAIL_Z) concentration. Data are displayed as mean value ±SD (n = 3 independent replicates). (D) Close-up view of the interaction site between the p300(BRPH_ΔAIL_Z) bromodomain and the nucleosomal DNA. (E) Electrophoretic mobility shift assay of the p300(BRPH_ΔAIL_Z) R1137A mutant with the NCP. Complex formation was analyzed by non-denaturing 4% polyacrylamide gel electrophoresis with SYBR Gold staining. Lanes 1–4 are control experiments with p300(BRPH_ΔAIL_Z), and lanes 5–8 are experiments with the p300(BRPH_ΔAIL_Z) R1137A mutant. (F) Quantitative results of the NCP-binding activity of the p300(BRPH_ΔAIL_Z) R1137A mutant. Ratios of the NCP bound to p300(BRPH_ΔAIL_Z) were estimated from the band intensity of the remaining free NCP, and the average values of three independent experiments (shown in panel (E) and Figure S5B) are plotted against the p300(BRPH_ΔAIL_Z) concentration. Data are displayed as mean value ±SD (n = 3 independent replicates).

The p300-NCP interface was further confirmed by mutagenesis. The basic patch on the surface of the HAT domain, composed of K1456, K1459, K1461, and R1462, is located near the nucleosomal DNA at the SHL2 site (Figure 3A), suggesting that these positively charged residues are involved in binding to the negatively charged DNA. Indeed, mutations of K1456, K1459, K1461, and R1462 to alanine substantially decreased the binding of p300(BRPH_ΔAIL_Z) to the NCP in electrophoretic mobility shift assays (Figures 3B, 3C, S1D, and S5A). Additionally, R1137 in the bromodomain of p300 is located close to the nucleosomal DNA at the SHL3 site (Figure 3D). The substitution of R1137 with alanine also led to a considerable reduction in the binding activity of p300(BRPH_ΔAIL_Z), judging from the presence of the strong free nucleosome band, although the bands corresponding to the p300(BRPH_ΔAIL_Z)-NCP complexes were still observed (Figures 3E and 3F, S1D, and S5B). Collectively, these results suggest that the DNA-binding functions of both the HAT domain and bromodomain are essential for the association of p300(BRPH_ΔAIL_Z) with the nucleosome.

### p300 binds to the nucleosome with various modes

**Figure 4:**
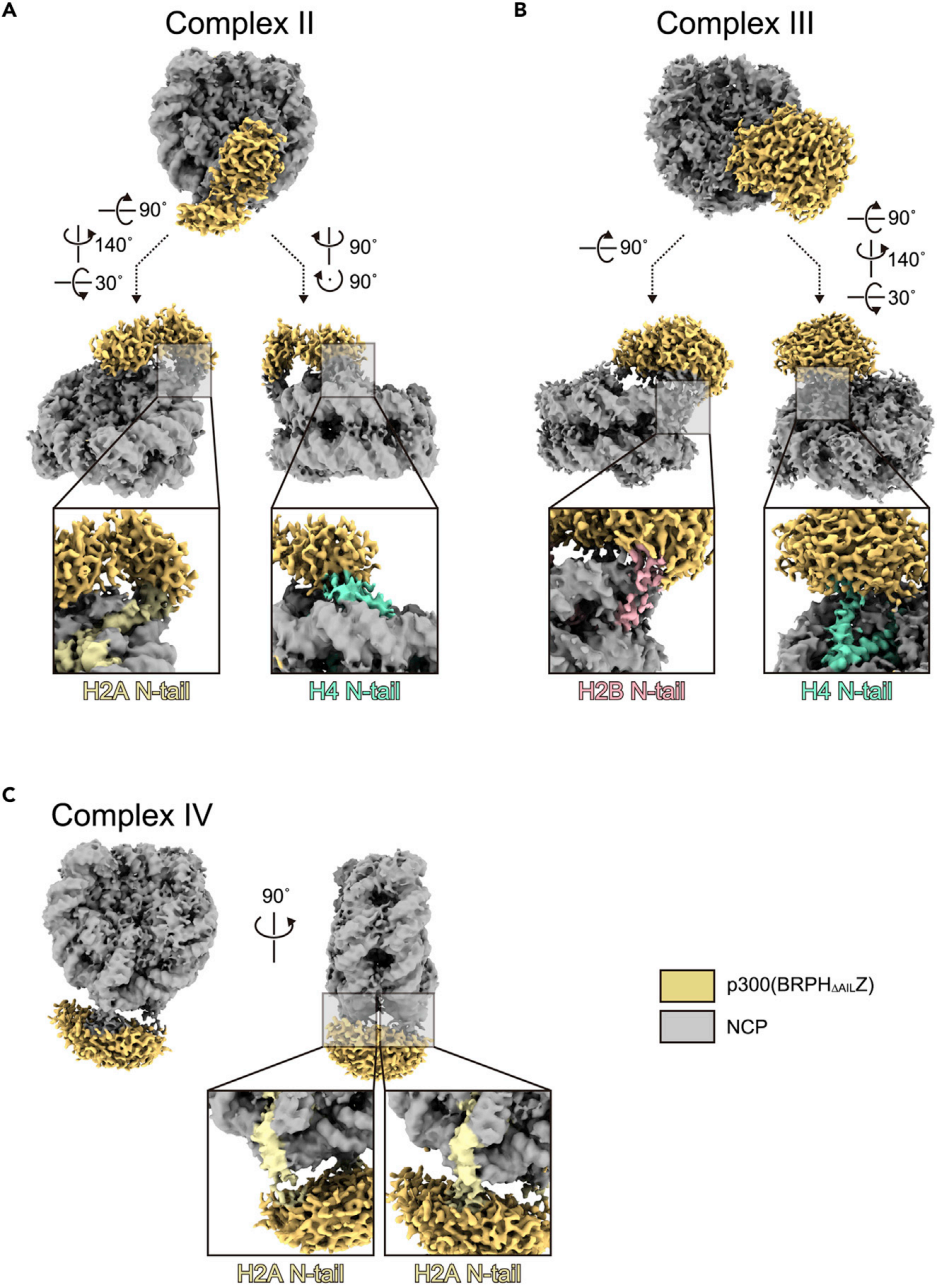
Cryo-EM structures of the p300(BRPH_ΔAIL_Z)-NCP complexes II, III, and IV (A) Cryo-EM map of the p300(BRPH_ΔAIL_Z)-NCP complex II. The densities corresponding to p300(BRPH_ΔAIL_Z) and NCP are yellow and gray, respectively. Enlarged views of the interaction sites between p300(BRPH_ΔAIL_Z) and the N-terminal tails of H2A and H4 are presented in the bottom panels. (B) Cryo-EM map of the p300(BRPH_ΔAIL_Z)-NCP complex III. Enlarged views of the interaction sites between p300(BRPH_ΔAIL_Z) and the N-terminal tails of H2B and H4 are presented in the bottom panels. (C) Cryo-EM map of the p300(BRPH_ΔAIL_Z)-NCP complex IV. Enlarged views of the interaction sites between p300(BRPH_ΔAIL_Z) and the two N-terminal tails of H2A are presented in the bottom panels.

Three additional classes of the p300(BRPH_ΔAIL_Z)-NCP structures, complex II, complex III, and complex IV, were obtained (Table 1). The cryo-EM density of p300(BRPH_ΔAIL_Z) in complex II is located on the side surface of the NCP, contacting the histone core and nucleosomal DNA (Figure 4A). The p300 density is also near the N-terminal tails of H2A and H4, suggesting that both tails could simultaneously engage either the bromodomain or the HAT domain in complex II (Figure 4A). Consequently, the complex II class structure apparently represents a p300 active form, in which the bromodomain recognizes a modified histone tail and mediates the acetylation of another histone tail by the HAT domain. Similarly, in complex III, the H2B and H4 N-terminal tails are incorporated within the p300(BRPH_ΔAIL_Z) density (Figure 4B). In contrast, in complex IV, the p300(BRPH_ΔAIL_Z) density is observed on the nucleosomal DNA, implying that p300 is bridging the neighboring DNA gyres and acting on the two N-terminal tails of H2A in the nucleosome (Figure 4C).

### Perspective

The p300-NCP complex structures described in this study reveal that p300 binds to the nucleosome via multiple binding modes, which allow p300 to prime and acetylate all four histone tails and various sites within these tails. The ability of p300 to adopt several conformations with respect to the nucleosome distinguishes this enzyme from the NuA4 HAT complex, which primarily acetylates H4 and therefore adopts a single conformation in the complex with the NCP (Xu et al., 2016). Future studies will focus on exploring additional modes for the association of p300 with post-translationally modified NCPs and the impact of DNA binding on p300 enzymatic activity.

### Limitations of the study

Resolutions of p300-NCP complex structures reported in the present study were not sufficient to reveal detailed p300-NCP interactions, which would explain how p300 promotes the acetylation of each nucleosomal histone tail and specifically recognizes the nucleosome containing post-translationally modified histones. In addition, the structure of p300 regions other than p300(BRPH_ΔAIL_Z) may also be important for a complete understanding of the mechanism by which p300 acetylates the nucleosomal histones.

## STAR★Methods

### Key resources table

**Table:** 

REAGENT or RESOURCE	SOURCE	IDENTIFIER
**Bacterial and virus strains**		

*E. coli* (BL21(DE3))	Merck	Cat#69450
*E. coli* (BL21(DE3))	Bio-Rad	Cat#156-3003
*E. coli* (JM109(DE3))	Promega	Cat#P9801
*E. coli* (BL21-CodonPlus (DE3)-RIL)	Agilent	Cat#230245
*E. coli* (DH5α)	Takara	Cat#9057

**Chemicals, peptides, and recombinant proteins**		

Recombinant human core histone H2A	Machida et al. (2018)	N/A
Recombinant human core histone H2B	Machida et al. (2018)	N/A
Recombinant human core histone H3.1	This paper	N/A
Recombinant human core histone H4	Machida et al. (2018)	N/A
Recombinant human p300(BRPH_ΔAIL_Z)	Zhang et al. (2018)	N/A
Recombinant human p300(BRPH_ΔAIL_Z) R1137A	This paper	N/A
Recombinant human p300(BRPH_ΔAIL_Z) BPD mutant (K1456A/K1459A/K1461A/R1462A)	This paper	N/A
Thrombin	Cytiva	Cat#27084601
Enterokinase, light chain	New England Biolabs	Cat#P8070L
Pre-Scission protease	Zhang et al. (2018)	N/A
Glutaraldehyde 25% solution, practical grade	Electron Microscopy Sciences	Cat# 16220-P
SYBR Gold	Thermo Fisher Scientific	Cat#S11494
DSS-H12/D12	Creative Molecules Inc.	Cat#001S
Trypsin/Lys-C Mix, Mass Spec Grade	Promega	Cat#V5072

**Deposited data**		

p300(BRPH_ΔAIL_Z)-NCP complex I	This paper	EMDB: EMD-32373 PDB: 7W9V
p300(BRPH_ΔAIL_Z)-NCP complex II	This paper	EMDB: EMD-32374
p300(BRPH_ΔAIL_Z)-NCP complex III	This paper	EMDB: EMD-32375
p300(BRPH_ΔAIL_Z)-NCP complex IV	This paper	EMDB: EMD-32376
Crystal structure of nucleosome	Vasudevan et al. (2010)	PDB: 3LZ0
Crystal structure of the p300 acetyltransferase catalytic core	Kaczmarska et al. (2017)	PDB: 5LKU
Crosslinking mass spectrometry data	This paper	jPOST: JPST001584 PXID: PXD033804
Mendeley Data	This paper	https://doi.org/10.17632/dgcnhyz779.1

**Recombinant DNA**		

pET-15b-H2A	Machida et al. (2018)	N/A
pET-15b-H2B	Machida et al. (2018)	N/A
pET-15b-H3.1 containing the enterokinase cleavage site	This paper	N/A
pET-15b-H4	Machida et al. (2018)	N/A
pGEX-6P-1-p300(BRPH_ΔAIL_Z)	Zhang et al. (2018)	N/A
pGEX-6P-1-p300(BRPH_ΔAIL_Z) R1137A	This paper	N/A
pGEX-6P-1-p300(BRPH_ΔAIL_Z) BPD mutant (K1456A/K1459A/K1461A/R1462A)	This paper	N/A
pGEM-T Easy-145 bp Widom 601 DNA	Arimura et al. (2013)	N/A

**Software and algorithms**		

SerialEM ver.3	Mastronarde (2005)	https://bio3d.colorado.edu/SerialEM/
Relion 3.1.2	Zivanov et al. (2018)	https://www3.mrc-lmb.cam.ac.uk/relion//index.php/Main_Page
MOTIONCOR2 1.4.0	Zheng et al. (2017)	https://emcore.ucsf.edu/ucsf-software
CTFFIND 4.1.14	Rohou and Grigorieff (2015)	http://grigoriefflab.janelia.org/ctf
UCSF ChimeraX-1.2.5	Goddard et al. (2018)	https://www.cgl.ucsf.edu/chimerax/
COOT 0.9.5	Emsley et al. (2010)	https://www2.mrc-lmb.cam.ac.uk/personal/pemsley/coot/
Phenix 1.19.2	Liebschner et al. (2019)	http://www.phenix-online.org
ISOLDE 1.2.2	Croll (2018)	https://isolde.cimr.cam.ac.uk/what-isolde/
MolProbity 4.5.1	Williams et al. (2018)	http://molprobity.biochem.duke.edu
xQuest/xProphet 2.1.3	Leitner et al. (2014)	https://gitlab.ethz.ch/leitner_lab/xquest_xprophet
ImageJ 1.52a	Schneider et al. (2012)	https://imagej.nih.gov/ij/

**Other**		

Ni-NTA agarose beads	QIAGEN	Cat#30250
Pierce Glutathione Agarose	Thermo Fisher Scientific	Cat#16101
Mono S HR 16/10 column	Cytiva	Cat#17050701
HiTrap Q HP anion exchange column	Cytiva	Cat#17115401
HiLoad 16/600 Superdex 75 pg column	Cytiva	Cat#28989333
HiLoad 16/600 Superdex 200 pg column	Cytiva	Cat#28989335
Superdex 30 Increase 3.2/300 column	Cytiva	Cat#29219758
PD-10 column	Cytiva	Cat#17085101
Pierce C18 Tips	Thermo Fisher Scientific	Cat# 87784
C18 NANO HPLC CAPILLARY COLUMN	Nikkyo Technos Co., Ltd.	Cat# NTCC-360/75-3-125
Amicon Ultra-4 centrifugal filter unit (30,000 MWCO)	Merck	Cat#UFC803096
Amicon Ultra-2 centrifugal filter unit (30,000 MWCO)	Merck	Cat#UFC203024
Peristaltic pump	ATTO Corporation	Cat#1221200
Model 491 Prep Cell	Bio-Rad	Cat#1702928
Q5 Site-Directed Mutagenesis Kit	New England Biolabs	Cat#E0554S
QuikChange Lightning Site-Directed Mutagenesis Kit	Agilent	Cat#210518
SW 41 Ti Swinging-Bucket Rotor	Beckman Coulter	Cat#331362
Quantifoil R1.2/1.3 200-mesh Cu	Quantifoil	Cat#M2955C-1
Vitrobot Mark IV	Thermo Fisher Scientific	
Krios G3i microscope	Thermo Fisher Scientific	
UltiMate 3000 UHPLC system	Thermo Fisher Scientific	
Orbitrap Fusion Tribrid mass spectrometer	Thermo Fisher Scientific	

### Resource availability

#### Lead contact

Further information and requests for resources and reagents should be directed to and will be fulfilled by the lead contact, Hitoshi Kurumizaka (kurumizaka@iqb.u-tokyo.ac.jp).

#### Materials availability

This study did not generate new unique reagents.

### Experimental model and subject details

Human histones H2A, H2B, and H3.1 were expressed in the *E. coli* BL21 (DE3) strain. Human histone H4 was expressed in the *E. coli* JM109 (DE3) strain. Human p300(BRPH_ΔAIL_Z) core proteins were expressed in the *E. coli* BL21-CodonPlus (DE3)-RIL strain.

### Method details

#### Histone purification

Human histones H2A, H2B, and H4 were bacterially produced and purified by the method described previously (Kujirai et al., 2018; Machida et al., 2018). The DNA fragment encoding human histone H3 was cloned into a modified pET-15b vector containing the His_6_-tag sequence and enterokinase cleavage site just upstream of the H3 sequence. The recombinant H3 protein was produced in *Escherichia coli* cells and purified by Ni-NTA column chromatography. To remove the His_6_-tag peptide, the resulting sample was treated with enterokinase (New England Biolabs) in buffer [20 mM Tris-HCl (pH 8.0), 50 mM NaCl, and 2 mM CaCl_2_]. Subsequent H3 purification steps were the same as those described previously (Kujirai et al., 2018).

#### Histone octamer reconstitution

Purified histones H2A, H2B, H3, and H4 were mixed in an equimolar ratio in denaturing buffer [20 mM Tris-HCl (pH 7.5), 7 M guanidine hydrochloride, and 20 mM 2-mercaptoethanol] and rotated at 4°C for 1.5 h. The histone octamer was assembled by dialysis against refolding buffer [10 mM Tris-HCl (pH 7.5), 2 M NaCl, 1 mM EDTA, and 5 mM 2-mercaptoethanol] at 4°C for 4 h, four times, and purified by size exclusion chromatography on a HiLoad 16/600 Superdex 200 prep grade column (Cytiva).

#### Nucleosome reconstitution

The DNA fragment containing the 145 base-pair Widom 601 sequence (Lowary and Widom, 1998) was prepared according to the previously reported method (Arimura et al., 2013; Dyer et al., 2004). Nucleosomes were reconstituted by the salt dialysis method (Kujirai et al., 2018) with slight modifications. The histone octamer and the DNA fragment were mixed (final DNA concentration, 0.8 mg/mL), and the sample was dialyzed against buffer [10 mM Tris-HCl (pH 7.5), 2 M KCl, 1 mM EDTA, and 1 mM dithiothreitol]. The KCl concentration in the buffer was gradually decreased to 250 mM using a peristaltic pump (ATTO Corporation). The sample was dialyzed against refolding buffer [10 mM Tris-HCl (pH 7.5), 250 mM KCl, 1 mM EDTA, and 1 mM dithiothreitol] at 4°C for 4 h. After dialysis, the resulting nucleosome was purified by 6% non-denaturing polyacrylamide gel electrophoresis in 0.5×TBE buffer [45 mM Tris-borate and 1 mM EDTA], using a Prep Cell apparatus (Bio-Rad). The nucleosome was concentrated with an Amicon Ultra-4 centrifugal filter unit (Merck, 30,000 MWCO) and dialyzed twice against final buffer [HEPES-NaOH (pH 7.5), 1 mM dithiothreitol, and 5% glycerol] at 4°C for 4 h. Purified nucleosomes were frozen in liquid nitrogen and stored at −80°C.

#### Purification of p300(BRPH_ΔAIL_Z)

The human p300(BRPH_ΔAIL_Z) (aa 1035–1720) core was cloned into the pGEX-6P-1 vector with an N-terminal GST tag and a Pre-Scission cleavage site. The flexible loop of residues 1520 to 1581 was replaced by an SGGSG linker, and the single mutation Y1467F was also introduced to stabilize the p300 core, based on former studies (Zhang et al., 2018). The histidine tag was added to the C-terminus of the core domain to increase the yield. The proteins were expressed in *E. coli* BL21-CodonPlus (DE3)-RIL competent cells in LB medium supplemented with 0.05 mM ZnCl_2_. Protein expression was induced with 0.5 mM IPTG for 20 h at 16°C. The proteins were purified with glutathione agarose in 50 mM Tris-HCl (pH 7.5) buffer, supplemented with 500 mM NaCl, 1 mM phenylmethanesulfonyl fluoride, and 5 mM β-mercaptoethanol. The GST tag was removed by overnight digestion at 4°C with Pre-Scission protease. The proteins were further purified by ion exchange on a HiTrap Q HP anion exchange column (Cytiva) and by size exclusion chromatography with a HiLoad 16/600 Superdex 75 column (Cytiva), in buffer [20 mM HEPES (pH 7.5) 200 mM NaCl, and 2 mM dithiothreitol]. All mutants were generated using either a QuikChange Site-Directed Mutagenesis Kit (Agilent) or a Q5 Site-Directed Mutagenesis Kit (New England Biolabs) according to the manufacturers’ protocols, and then grown and purified as described above.

#### Assay for p300(BRPH_ΔAIL_Z)-NCP binding

The NCP (10 nM) and p300(BRPH_ΔAIL_Z) or p300(BRPH_ΔAIL_Z) mutants (0.4, 0.8, and 1.2 μM) were incubated at 25°C for 30 min in buffer [20 mM HEPES-NaOH (pH 7.5), 40 mM NaCl, 1 mM MgCl_2_, 1 μM Zn(OAc)_2_, 1.1 mM dithiothreitol, 0.03% NP-40, and 0.5% glycerol]. Afterwards, 1% glutaraldehyde was added to a final concentration of 0.1%, and the reaction was incubated at 4°C for 30 min. The crosslinking was quenched by the addition of 1 M Tris-HCl (pH 7.5) to a final concentration of 50 mM, and the reaction was incubated on ice for 10 min. The samples were analyzed by 4% non-denaturing polyacrylamide gel electrophoresis in 0.5×TBE buffer [45 mM Tris-borate and 1 mM EDTA], followed by SYBR Gold staining. Ratios of the NCP bound to p300(BRPH_ΔAIL_Z) were calculated as follows: F(*x*) = [G(0)-G(*x*)]/[G(0)]. Here G(0) is the band intensities of the experiments without the p300(BRPH_ΔAIL_Z) protein and G(*x*) is the band intensities of the experiments with each concentration of the p300(BRPH_ΔAIL_Z) protein.

#### Assay for p300(BRPH_ΔAIL_Z)-DNA binding

The naked 145 base-pair Widom 601 DNA (10 nM) and p300(BRPH_ΔAIL_Z) (0.4, 0.8, and 1.2 μM) were incubated at 25°C for 30 min in buffer [20 mM HEPES-NaOH (pH 7.5), 40 mM NaCl, 1 mM MgCl_2_, 1 μM Zn(OAc)_2_, 1.1 mM dithiothreitol, 0.03% NP-40, and 0.5% glycerol]. Afterwards, the samples were analyzed by 4% non-denaturing polyacrylamide gel electrophoresis in 0.5×TBE buffer [45 mM Tris-borate and 1 mM EDTA], followed by SYBR Gold staining.

#### Preparation of p300(BRPH_ΔAIL_Z)-NCP complex for cryo-EM analysis

The NCP (0.1 μM) and p300(BRPH_ΔAIL_Z) (2 μM) were incubated at 25°C for 30 min in buffer [20 mM HEPES-NaOH (pH 7.5), 20 mM NaCl, 0.5 mM MgCl_2_, 1 μM Zn(OAc)_2_, 1 mM dithiothreitol, 0.03% NP-40, and 0.5% glycerol]. The sample was then crosslinked by adding 2.5% glutaraldehyde to a final concentration of 0.1%, and incubated at 4°C for 30 min. The crosslinking reaction was quenched by adding 1 M Tris-HCl (pH 7.5) to a final concentration of 50 mM and incubating it on ice for 10 min. The crosslinked sample was applied onto the top of a sucrose density gradient [5–20% sucrose gradient in 10 mM Tris-HCl (pH 7.5), 30 mM NaCl, 1 μM Zn(OAc)_2_, and 1 mM dithiothreitol] and centrifugated at 27,000 r.p.m., at 4°C for 16 h in an SW 41 Ti rotor (Beckman Coulter). After the ultracentrifugation, aliquots (630 μL) were collected from the top of the gradient and analyzed by 4% non-denaturing polyacrylamide gel electrophoresis in 0.5×TBE buffer, followed by SYBR Gold staining. The fractions containing the p300(BRPH_ΔAIL_Z)-NCP complexes were combined, and then desalted using a PD-10 column (Cytiva) in final buffer [10 mM Tris-HCl (pH 7.5), 30 mM NaCl, and 1 mM dithiothreitol]. The sample was then concentrated with an Amicon Ultra-2 centrifugal filter unit (Merck, 30,000 MWCO) and stored on ice.

#### Cryo-EM grid preparation and data collection

Aliquots (2.5 μL) of the purified p300(BRPH_ΔAIL_Z)-NCP complex were applied to glow-discharged grids (Quantifoil R1.2/1.3 200-mesh Cu). The grids were blotted for 8 s under 100% humidity at 4°C, using a Vitrobot Mark IV (Thermo Fisher Scientific), and plunged into liquid ethane. Cryo-EM data of the p300(BRPH_ΔAIL_Z)-NCP complexes were collected using the SerialEM auto acquisition software (Mastronarde, 2005) on a Krios G3i microscope (Thermo Fisher Scientific), operating at 300 kV at a magnification of 81,000× (pixel size of 1.07 Å) with an energy-filtered K3 detector. Digital micrographs were recorded with a 10-s exposure time in the electron counting mode and defocus ranging from −1.2 to −2.3 μm on a Falcon 3 direct detector (Thermo Fisher Scientific), retaining a stack of 40 frames with an accumulated total dose of 55.975 electrons per Å^2^ in the 1st dataset collection and 56.149 electrons per Å^2^ in the 2nd dataset collection.

#### Image processing

Details of the image processing are provided in Figure S3 and Table 1. All frames in movies from the 1st and 2nd datasets were aligned by MOTIONCOR2 (Zheng et al., 2017), with dose weighting. The contrast transfer function (CTF) was estimated using CTFFIND4 (Rohou and Grigorieff, 2015) from digital micrographs, and micrographs were selected based on good CTF fit correlation. The subsequent image processing was performed with Relion 3.1 (Zivanov et al., 2018). After automatically picking particles from the micrographs, 2D classification was performed three times to discard junk particles, and selected particles were subjected to the following 3D classification. The crystal structure of a canonical nucleosome (PDB: 3LZ0) (Vasudevan et al., 2010) was used as the initial alignment model with low-pass filtering of 60 Å. After the first 3D classification, selected particles from the two datasets were joined and subjected to the next rounds of 3D classification. The particles in the suitable classes were selected and subjected to 3D auto-refinement with masking of the nucleosome, followed by 3D classification without image alignment. The best 3D class with extra density of p300(BRPH_ΔAIL_Z) was selected and used as the reference model for the following 3D classification. In the next 3D classification, the p300(BRPH_ΔAIL_Z)-NCP complexes II and IV were obtained, and the 3D class with densities in the same positions as the reference was subjected to the following 3D classification. In the next 3D classification, the p300(BRPH_ΔAIL_Z)-NCP complex III was obtained, and the 3D class with densities in the same position as the reference was subjected to 3D auto-refinement and post-processing, followed by Bayesian polishing and CTF refinement. The 3D class was subjected to 3D auto-refinement and post-processing again, and the final cryo-EM map of the p300(BRPH_ΔAIL_Z)-NCP complex I was obtained. The resolution of the refined 3D map of the p300(BRPH_ΔAIL_Z)-NCP complex I was estimated at 3.95 Å by the “gold standard” Fourier Shell Correlation (FSC) at an FSC = 0.143 (Scheres, 2016). The cryo-EM maps of the p300(BRPH_ΔAIL_Z)-NCP complex I-IV were ad-hoc low-pass filtered at 4.0 Å. The figures of the p300(BRPH_ΔAIL_Z)-NCP complexes I-IV were created by UCSF ChimeraX (Goddard et al., 2018) with the “Hide dust” tool for removing noisy densities.

#### Model building

Crystal structures of an NCP containing the Widom 601 DNA and *Xenopus laevis* histones (PDB: 3LZ0) (Rohou and Grigorieff, 2015) and the p300 acetyltransferase catalytic core with coenzyme A (PDB: 5LKU) (Kaczmarska et al., 2017) were used for the model building of the p300(BRPH_ΔAIL_Z)-NCP complex I. The amino acid residues of the NCP were replaced with those of human histones by using COOT (Emsley et al., 2010). The crystal structure of the NCP was manually fitted into the cryo-EM density map of the p300(BRPH_ΔAIL_Z)-NCP complex I, and was positionally refined by rigid body optimization with UCSF ChimeraX (Goddard et al., 2018). The atomic coordinates of the NCP were refined using phenix_real_space_refine (Liebschner et al., 2019), followed by manual editing with interactive molecular dynamics flexible fitting using ISOLDE (Croll, 2018). The crystal structure of the p300 acetyltransferase catalytic core was manually fitted into the cryo-EM density map of the p300(BRPH_ΔAIL_Z)-NCP complex I with UCSF ChimeraX (Goddard et al., 2018). The final model of the p300(BRPH_ΔAIL_Z)-NCP complex I was evaluated by MolProbity (Williams et al., 2018) (Table 1). Structural figures were created with UCSF ChimeraX (Goddard et al., 2018).

#### Crosslinking mass spectrometry

p300(BRPH_ΔAIL_Z) (5 μM) was mixed with the NCP (0.25 μM) in reaction buffer [20 mM HEPES-NaOH (pH 7.5), 20 mM NaCl, 0.5 mM MgCl_2_, 1 μM Zn(OAc)_2_, 1 mM dithiothreitol, 0.03% NP-40, and 0.5% glycerol] at 25°C for 30 min. After this incubation, the sample was crosslinked with 800 μM DSS-H12/D12 at 25°C for 30 min. The crosslinking reaction was quenched by the addition of 50 mM Tris-HCl (pH 7.5) and incubated at 25°C for 30 min. The sample was dried, and the residue was dissolved in an 8 M urea solution to a 1 mg/mL final protein concentration. The crosslinked proteins were reduced by an incubation with 2.5 mM TCEP for 30 min at 37°C, and further alkylated by an incubation with 5 mM iodoacetamide for 30 min at room temperature with light shielding. This sample was diluted to a final concentration of 1 M urea in a solution containing 50 mM ammonium bicarbonate, and digested with Trypsin/Lys-C Mix, Mass Spec Grade (Promega) at 37°C overnight (1:50 wt/wt enzyme to substrate ratio). The digestion was stopped by adding 5% (vol/vol) trifluoroacetic acid (TFA). All peptides were purified by Pierce C18 Tips (Thermo Fisher Scientific) eluted with 25, 50, and 80% acetonitrile. The eluted samples were dried, and the residue was dissolved in water/acetonitrile/TFA (75:25:0.1). The crosslinked peptide aliquot (50 μL) was fractionated on a Superdex 30 Increase 3.2/300 column (Cytiva) in water/acetonitrile/TFA (75:25:0.1), at a flow rate of 50 μL/min. Fractions (100 μL) were collected, dried, and redissolved in 0.1% TFA for analysis by liquid chromatography-tandem mass spectrometry (LC-MS/MS). The analysis was performed using an Orbitrap Fusion Tribrid mass spectrometer equipped with an UltiMate 3000 UHPLC system (Thermo Fisher Scientific). A C18 NANO HPLC CAPILLARY COLUMN (Nikkyo Technos Co., Ltd.) of fully porous particles (particle size 3 μm, inner diameter 75 μm, length 125 mm) was used for nano-LC, and the mobile phase was a linear gradient expanded from 5% to 50% acetonitrile at 300 nL/min for 90 min. The precursor ions were acquired in the 350-1,500 Da mass range, with a resolution of 60,000 full widths at half maximum. In the data-dependent scan, precursors with 2-7 charges were selected for the MS/MS scan. The selected ions were sequentially isolated and fragmented by collision-induced dissociation. The cross-linked peptides were identified by xQuest, and the false discovery rate (FDR) was estimated by xProphet (Leitner et al., 2014). The results from xProphet were filtered according to the following parameters: FDR <0.05, minimum δ-score = 0.85, minimum border of MS1 tolerance = −4 ppm, maximum border of MS1 tolerance = 7 ppm, ld-score > 10. The cross-linking between p300(BRPH_ΔAIL_Z) and NCP was visualized by the webserver xVis (Grimm et al., 2015).

### Quantification and statistical analysis

In Figures 3C–3F, the band intensities of the remaining free NCPs were quantified using ImageJ (Schneider et al., 2012), and the average values of three independent experiments are shown with the SD values. The experiment shown in Figure S1C was performed twice, and reproducible results were obtained.

## Data Availability

The cryo-EM reconstructions of the p300(BRPH_ΔAIL_Z)-NCP complexes have been deposited in the Electron Microscopy DataBank, and the atomic model of the p300(BRPH_ΔAIL_Z)-NCP complex I has been deposited in the Protein Data Bank, under the accession codes (EMD-32373 and PDB ID 7W9V for complex I; and EMD-32374, EMD-32375, and EMD-32376 for complexes II, III, and IV, respectively). Crosslinking mass spectrometry data used in this study have been deposited in the proteomeXchange Consortium (PXD033804) via the Japan ProteOme STandard Repository (JPST001584). Original gel images have been deposited to Mendeley Data (https://doi.org/10.17632/dgcnhyz779.1). This paper does not report original code. Any additional information required to reanalyze the data reported in this paper is available from the lead contact upon request.

## References

[bib1] An W., Kim J., Roeder R.G. (2004). Ordered cooperative functions of PRMT1, p300, and CARM1 in transcriptional activation by p53. Cell.

[bib2] Arimura Y., Kimura H., Oda T., Sato K., Osakabe A., Tachiwana H., Sato Y., Kinugasa Y., Ikura T., Sugiyama M. (2013). Structural basis of a nucleosome containing histone H2A.B/H2A.Bbd that transiently associates with reorganized chromatin. Sci. Rep..

[bib3] Bose D.A., Donahue G., Reinberg D., Shiekhattar R., Bonasio R., Berger S.L. (2017). RNA binding to CBP stimulates histone acetylation and transcription. Cell.

[bib4] Croll T.I. (2018). ISOLDE: a physically realistic environment for model building into low-resolution electron-density maps. Acta Crystallogr. D Struct. Biol..

[bib5] Dancy B.M., Cole P.A. (2015). Protein lysine acetylation by p300/CBP. Chem. Rev..

[bib6] Demarest S.J., Martinez-Yamout M., Chung J., Chen H., Xu W., Dyson H.J., Evans R.M., Wright P.E. (2002). Mutual synergistic folding in recruitment of CBP/p300 by p160 nuclear receptor coactivators. Nature.

[bib7] Dyer P.N., Edayathumangalam R.S., White C.L., Bao Y., Chakravarthy S., Muthurajan U.M., Luger K. (2004). Reconstitution of nucleosome core particles from recombinant histones and DNA. Methods Enzymol..

[bib8] Emsley P., Lohkamp B., Scott W.G., Cowtan K. (2010). Features and development of Coot. Acta Crystallogr. D Biol. Crystallogr..

[bib9] Goddard T.D., Huang C.C., Meng E.C., Pettersen E.F., Couch G.S., Morris J.H., Ferrin T.E. (2018). UCSF ChimeraX: meeting modern challenges in visualization and analysis. Protein Sci..

[bib10] Grimm M., Zimniak T., Kahraman A., Herzog F. (2015). xVis: a web server for the schematic visualization and interpretation of crosslink-derived spatial restraints. Nucleic Acids Res..

[bib11] Gu W., Roeder R.G. (1997). Activation of p53 sequence-specific DNA binding by acetylation of the p53 C-terminal domain. Cell.

[bib12] Hasan S., Hassa P.O., Imhof R., Hottiger M.O. (2001). Transcription coactivator p300 binds PCNA and may have a role in DNA repair synthesis. Nature.

[bib13] Iyer N.G., Özdag H., Caldas C. (2004). p300/CBP and cancer. Oncogene.

[bib14] Jin Q., Yu L.R., Wang L., Zhang Z., Kasper L.H., Lee J.E., Wang C., Brindle P.K., Dent S.Y.R., Ge K. (2011). Distinct roles of GCN5/PCAF-mediated H3K9ac and CBP/p300-mediated H3K18/27ac in nuclear receptor transactivation. EMBO J..

[bib15] Kaczmarska Z., Ortega E., Goudarzi A., Huang H., Kim S., Márquez J.A., Zhao Y., Khochbin S., Panne D. (2017). Structure of p300 in complex with acyl-CoA variants. Nat. Chem. Biol..

[bib16] Kouzarides T. (2007). Chromatin modifications and their function. Cell.

[bib17] Kujirai T., Arimura Y., Fujita R., Horikoshi N., Machida S., Kurumizaka H. (2018). Methods for preparing nucleosomes containing histone variants. Methods Mol. Biol..

[bib18] Lai W.K.M., Pugh B.F. (2017). Understanding nucleosome dynamics and their links to gene expression and DNA replication. Nat. Rev. Mol. Cell Biol..

[bib19] Lasko L.M., Jakob C.G., Edalji R.P., Qiu W., Montgomery D., Digiammarino E.L., Hansen T.M., Risi R.M., Frey R., Manaves V. (2017). Discovery of a selective catalytic p300/CBP inhibitor that targets lineage-specific tumours. Nature.

[bib20] Leitner A., Walzthoeni T., Aebersold R. (2014). Lysine-specific chemical cross-linking of protein complexes and identification of cross-linking sites using LC-MS/MS and the xQuest/xProphet software pipeline. Nat. Protoc..

[bib21] Liebschner D., Afonine P.V., Baker M.L., Bunkóczi G., Chen V.B., Croll T.I., Hintze B., Hung L.W., Jain S., McCoy A.J. (2019). Macromolecular structure determination using X-rays, neutrons and electrons: recent developments in Phenix. Acta Crystallogr. D Struct. Biol..

[bib22] Liu X., Wang L., Zhao K., Thompson P.R., Hwang Y., Marmorstein R., Cole P.A. (2008). The structural basis of protein acetylation by the p300/CBP transcriptional coactivator. Nature.

[bib23] Lowary P.T., Widom J. (1998). New DNA sequence rules for high affinity binding to histone octamer and sequence-directed nucleosome positioning. J. Mol. Biol..

[bib24] Luger K., Mäder A.W., Richmond R.K., Sargent D.F., Richmond T.J. (1997). Crystal structure of the nucleosome core particle at 2.8 Å resolution. Nature.

[bib25] Machida S., Takizawa Y., Ishimaru M., Sugita Y., Sekine S., Nakayama J.I., Wolf M., Kurumizaka H. (2018). Structural basis of heterochromatin formation by human HP1. Mol. Cell.

[bib26] Mastronarde D.N. (2005). Automated electron microscope tomography using robust prediction of specimen movements. J. Struct. Biol..

[bib27] Musselman C.A., Lalonde M.E., Côté J., Kutateladze T.G. (2012). Perceiving the epigenetic landscape through histone readers. Nat. Struct. Mol. Biol..

[bib28] Ogryzko V.V., Schiltz R.L., Russanova V., Howard B.H., Nakatani Y. (1996). The transcriptional coactivators p300 and CBP are histone acetyltransferases. Cell.

[bib29] Rohou A., Grigorieff N. (2015). CTFFIND4: Fast and accurate defocus estimation from electron micrographs. J. Struct. Biol..

[bib30] Scheres S.H.W. (2016). Processing of structurally heterogeneous cryo-EM data in RELION. Methods Enzymol..

[bib31] Schiltz R.L., Mizzen C.A., Vassilev A., Cook R.G., Allis C.D., Nakatani Y. (1999). Overlapping but distinct patterns of histone acetylation by the human coactivators p300 and PCAF within nucleosomal substrates. J. Biol. Chem..

[bib32] Schneider C.A., Rasband W.S., Eliceiri K.W. (2012). NIH Image to ImageJ: 25 years of image analysis. Nat. Methods.

[bib33] Shin M.K., Vázquez-Rosa E., Koh Y., Dhar M., Chaubey K., Cintrón-Pérez C.J., Barker S., Miller E., Franke K., Noterman M.F. (2021). Reducing acetylated tau is neuroprotective in brain injury. Cell.

[bib34] Tang Z., Chen W.Y., Shimada M., Nguyen U.T.T., Kim J., Sun X.J., Sengoku T., McGinty R.K., Fernandez J.P., Muir T.W., Roeder R.G. (2013). SET1 and p300 act synergistically, through coupled histone modifications, in transcriptional activation by p53. Cell.

[bib35] Tessarz P., Kouzarides T. (2014). Histone core modifications regulating nucleosome structure and dynamics. Nat. Rev. Mol. Cell Biol..

[bib36] Teves S.S., Weber C.M., Henikoff S. (2014). Transcribing through the nucleosome. Trends Biochem. Sci..

[bib37] Thompson P.R., Kurooka H., Nakatani Y., Cole P.A. (2001). Transcriptional coactivator protein p300. J. Biol. Chem..

[bib38] Thompson P.R., Wang D., Wang L., Fulco M., Pediconi N., Zhang D., An W., Ge Q., Roeder R.G., Wong J. (2004). Regulation of the p300 HAT domain via a novel activation loop. Nat. Struct. Mol. Biol..

[bib39] Turnell A.S., Stewart G.S., Grand R.J.A., Rookes S.M., Martin A., Yamano H., Elledge S.J., Gallimore P.H. (2005). The APC/C and CBP/p300 cooperate to regulate transcription and cell-cycle progression. Nature.

[bib40] Vasudevan D., Chua E.Y.D., Davey C.A. (2010). Crystal structures of nucleosome core particles containing the “601” strong positioning sequence. J. Mol. Biol..

[bib41] Wang F., Marshall C.B., Ikura M. (2013). Transcriptional/epigenetic regulator CBP/p300 in tumorigenesis: structural and functional versatility in target recognition. Cell. Mol. Life Sci..

[bib42] Weinert B.T., Narita T., Satpathy S., Srinivasan B., Hansen B.K., Schölz C., Hamilton W.B., Zucconi B.E., Wang W.W., Liu W.R. (2018). Time-resolved analysis reveals rapid dynamics and broad scope of the CBP/p300 acetylome. Cell.

[bib43] Williams C.J., Headd J.J., Moriarty N.W., Prisant M.G., Videau L.L., Deis L.N., Verma V., Keedy D.A., Hintze B.J., Chen V.B. (2018). MolProbity: more and better reference data for improved all-atom structure validation. Protein Sci..

[bib44] Xu P., Li C., Chen Z., Jiang S., Fan S., Wang J., Dai J., Zhu P., Chen Z. (2016). The NuA4 core complex acetylates nucleosomal histone H4 through a double recognition mechanism. Mol. Cell.

[bib45] Zentner G.E., Henikoff S. (2013). Regulation of nucleosome dynamics by histone modifications. Nat. Struct. Mol. Biol..

[bib46] Zhang Y., Xue Y., Shi J., Ahn J.W., Mi W., Ali M., Wang X., Klein B.J., Wen H., Li W. (2018). The ZZ domain of p300 mediates specificity of the adjacent HAT domain for histone H3. Nat. Struct. Mol. Biol..

[bib47] Zheng S.Q., Palovcak E., Armache J.P., Verba K.A., Cheng Y., Agard D.A. (2017). MotionCor2: anisotropic correction of beam-induced motion for improved cryo-electron microscopy. Nat. Methods.

[bib48] Zivanov J., Nakane T., Forsberg B.O., Kimanius D., Hagen W.J.H., Lindahl E., Scheres S.H.W. (2018). New tools for automated high-resolution cryo-EM structure determination in RELION-3. Elife.

