# Supplementary material for: Structural basis for binding diversity of acetyltransferase p300 to the nucleosome
Source: iScience. 2022 Jun 9;25(7):104563. doi: 10.1016/j.isci.2022.104563 (PMC9218434; doi:10.1016/j.isci.2022.104563)
Supplement: Document S1. Figures S1–S5 [file mmc1.pdf]

iScience, Volume *25*

Supplemental information

Structural basis for binding diversity

# of acetyltransferase p300 to the nucleosome

Suguru Hatazawa, Jiuyang Liu, Yoshimasa Takizawa, Mohamad Zandian, Lumi Negishi, Tatiana G. Kutateladze, and Hitoshi Kurumizaka

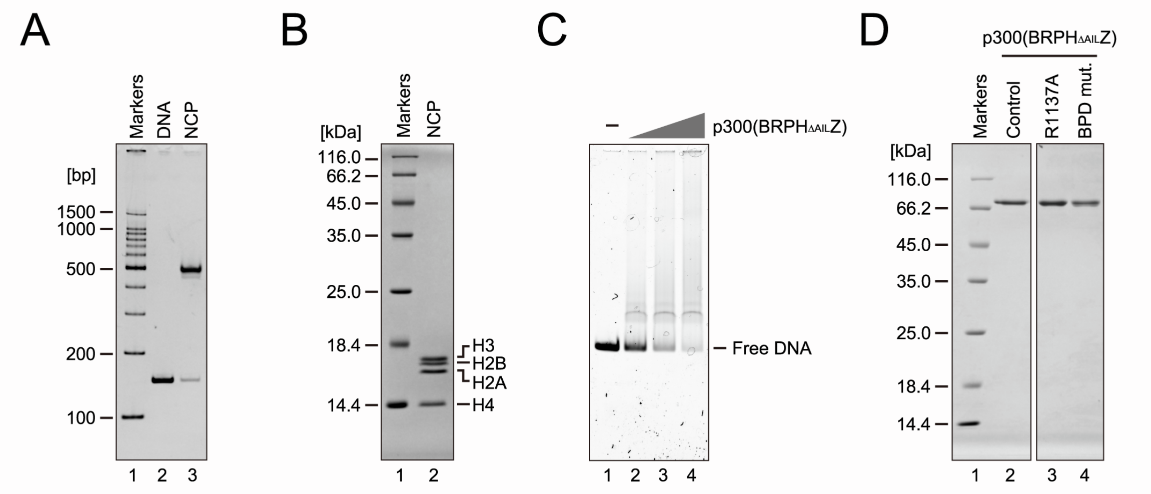

Figure S1. Preparation of the NCP and the p300(BRPH$_{\Delta AIL}$Z) proteins and assay for p300(BRPH$_{\Delta AIL}$Z)-DNA binding, related to Figures 1 and 3. (A) The 145 base-pair Widom 601 DNA and the purified NCP containing the DNA were analyzed by non-denaturing 6% polyacrylamide gel electrophoresis with ethidium bromide staining. (B) The nucleosomal histones were analyzed by 18% SDS-polyacrylamide gel electrophoresis with Coomassie Brilliant Blue staining. (C) Electrophoretic mobility shift assay of p300(BRPH$_{\Delta AIL}$Z) and the naked 145 base-pair Widom 601 DNA. The binding of p300(BRPH$_{\Delta AIL}$Z) and DNA was analyzed by non-denaturing 4% polyacrylamide gel electrophoresis with SYBR Gold staining. (D) The purified p300(BRPH$_{\Delta AIL}$Z) proteins were analyzed by 12% SDS-polyacrylamide gel electrophoresis with Coomassie Brilliant Blue staining.

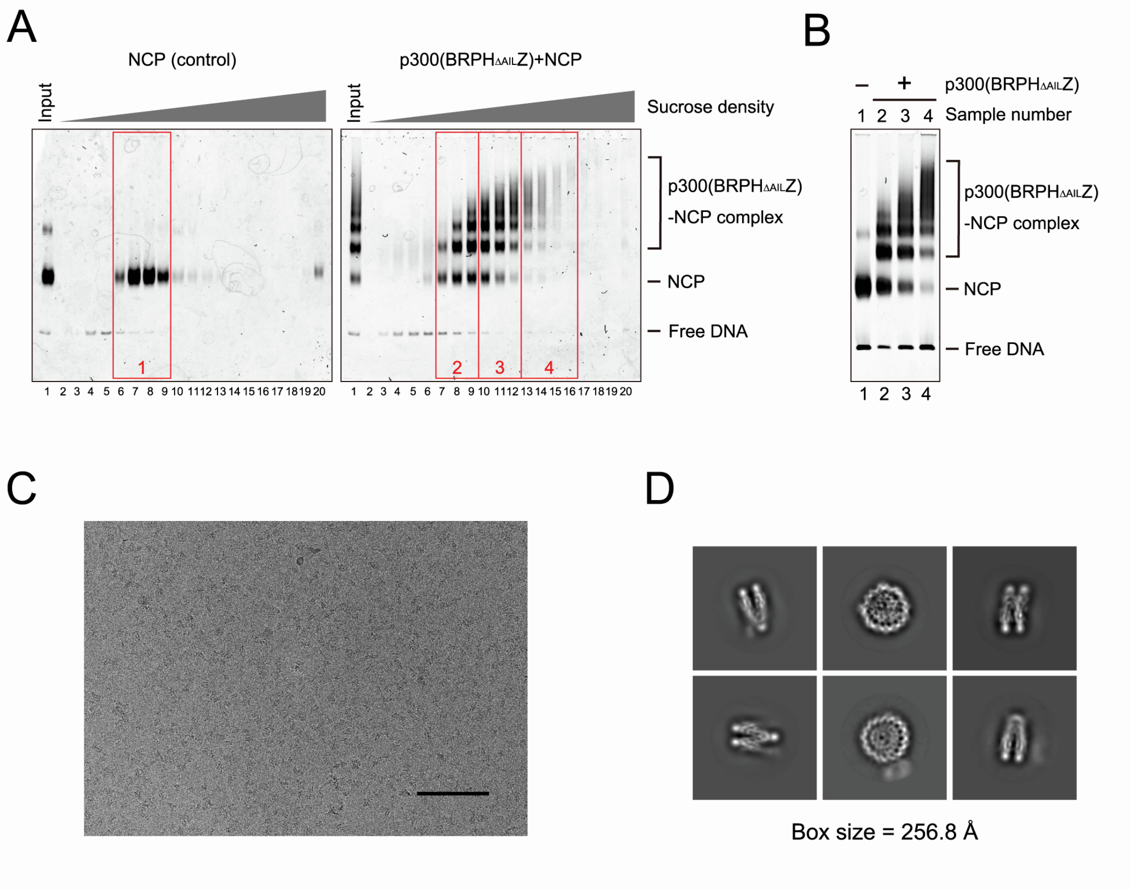

Figure S2. Preparation and cryo-EM analysis of the p300(BRPH$_{\Delta AIL}$Z)-NCP complexes, related to Figures 2 and 4. (A) The p300(BRPH$_{\Delta AIL}$Z)-NCP complexes were purified by sucrose density gradient centrifugation, and the collected fractions were analyzed by non-denaturing 4% polyacrylamide gel electrophoresis with SYBR Gold staining. The fractions shown in the red frames were combined. (B) The purified NCP and p300(BRPH$_{\Delta AIL}$Z)-NCP samples were analyzed by non-denaturing 4% polyacrylamide gel electrophoresis with SYBR Gold staining. Sample number 3 was used for the cryo-EM analysis. (C) A representative image from a digital cryo-EM micrograph of the p300(BRPH$_{\Delta AIL}$Z)-NCP complexes. Scale bar indicates 100 nm. (D) Representative 2D class averages of the p300(BRPH$_{\Delta AIL}$Z)-NCP complex from single particle images.

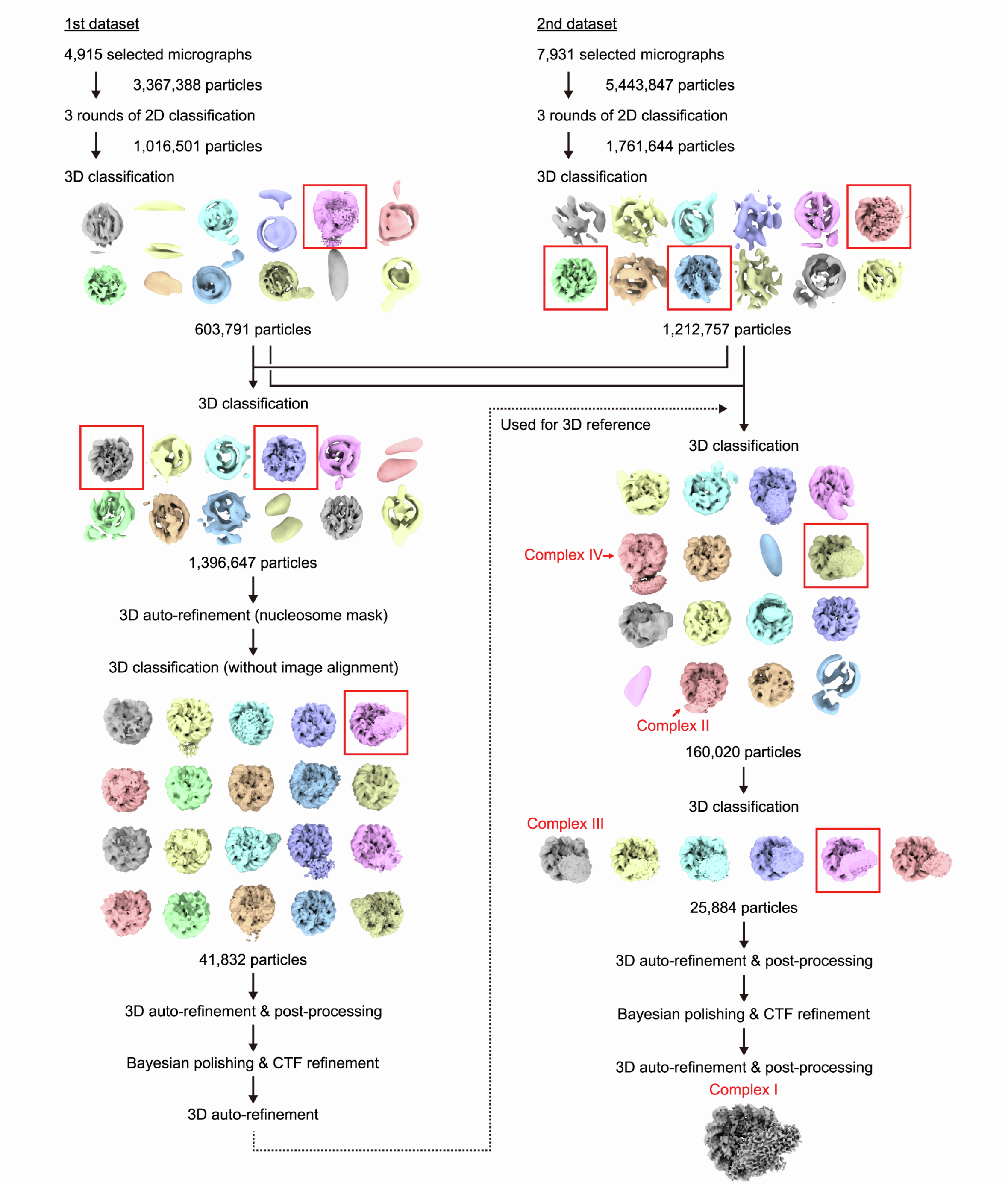

Figure S3. Workflow for cryo-EM processing of the p300(BRPH$_{\Delta AIL}$Z)-NCP complex related to Figures 2 and 4. The particles contained in the classes enclosed by the red squares were used in the subsequent processes.

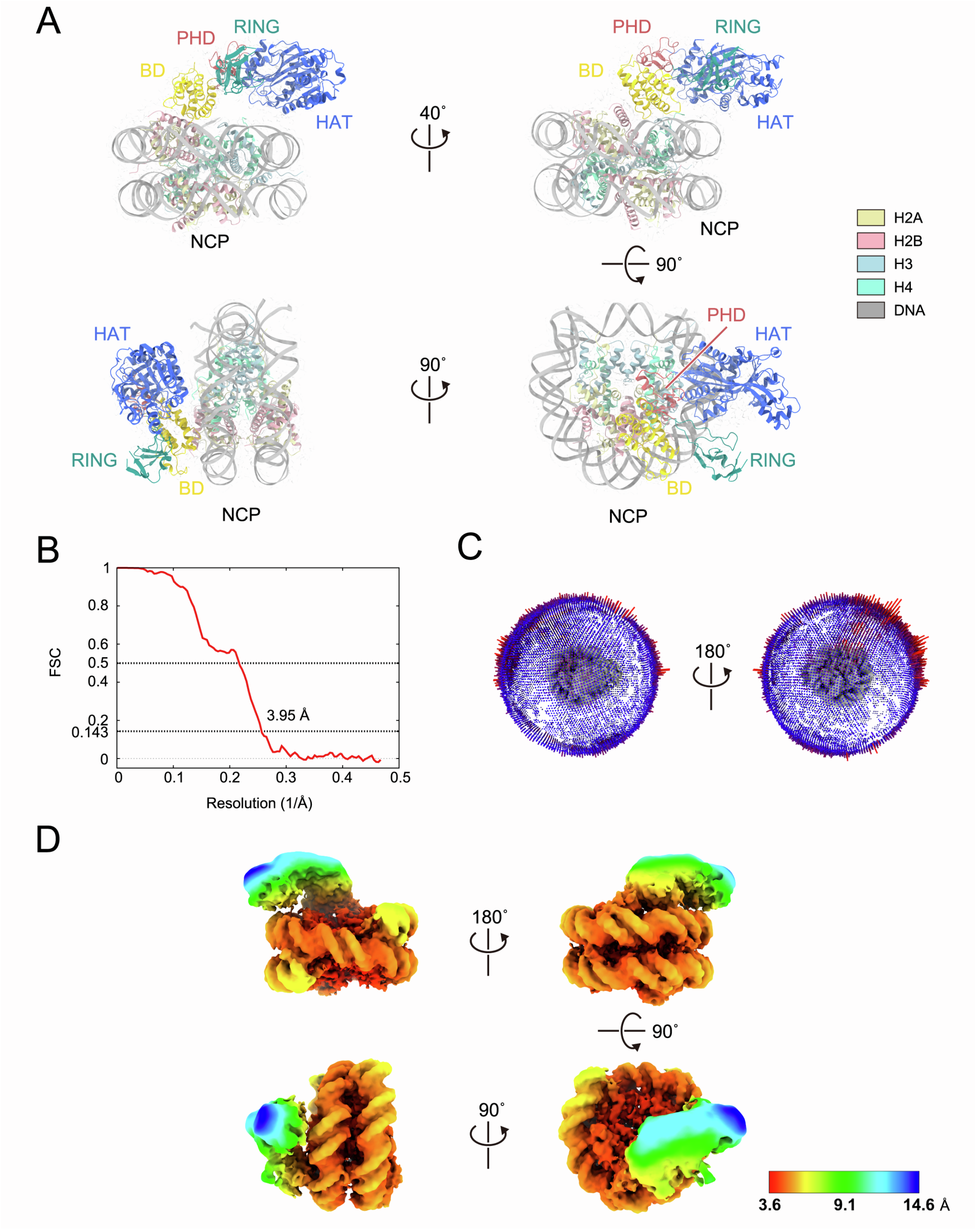

Figure S4. Cryo-EM structure of the p300(BRPH$_{\Delta AIL}$Z)-NCP complex I, related to Figure 2. (A)

The overall structure of the p300(BRPH$_{\Delta AIL}$Z)-NCP complex I. The crystal structures of the NCP and

the p300 catalytic core are superimposed on the cryo-EM map. (B) Gold-standard Fourier Shell Correlation (FSC) curve of the p300(BRPH$_{\Delta AIL}$Z)-NCP complex I. Its overall resolution is 3.95 Å at FSC = 0.143. (C) Angular distribution of model projections of the p300(BRPH$_{\Delta AIL}$Z)-NCP complex I. (D) Local resolution map of the p300(BRPH$_{\Delta AIL}$Z)-NCP complex I estimated by the RELION 3.1 postprocess, showing the resolution range across the map from 3.6 Å to 14.6 Å.

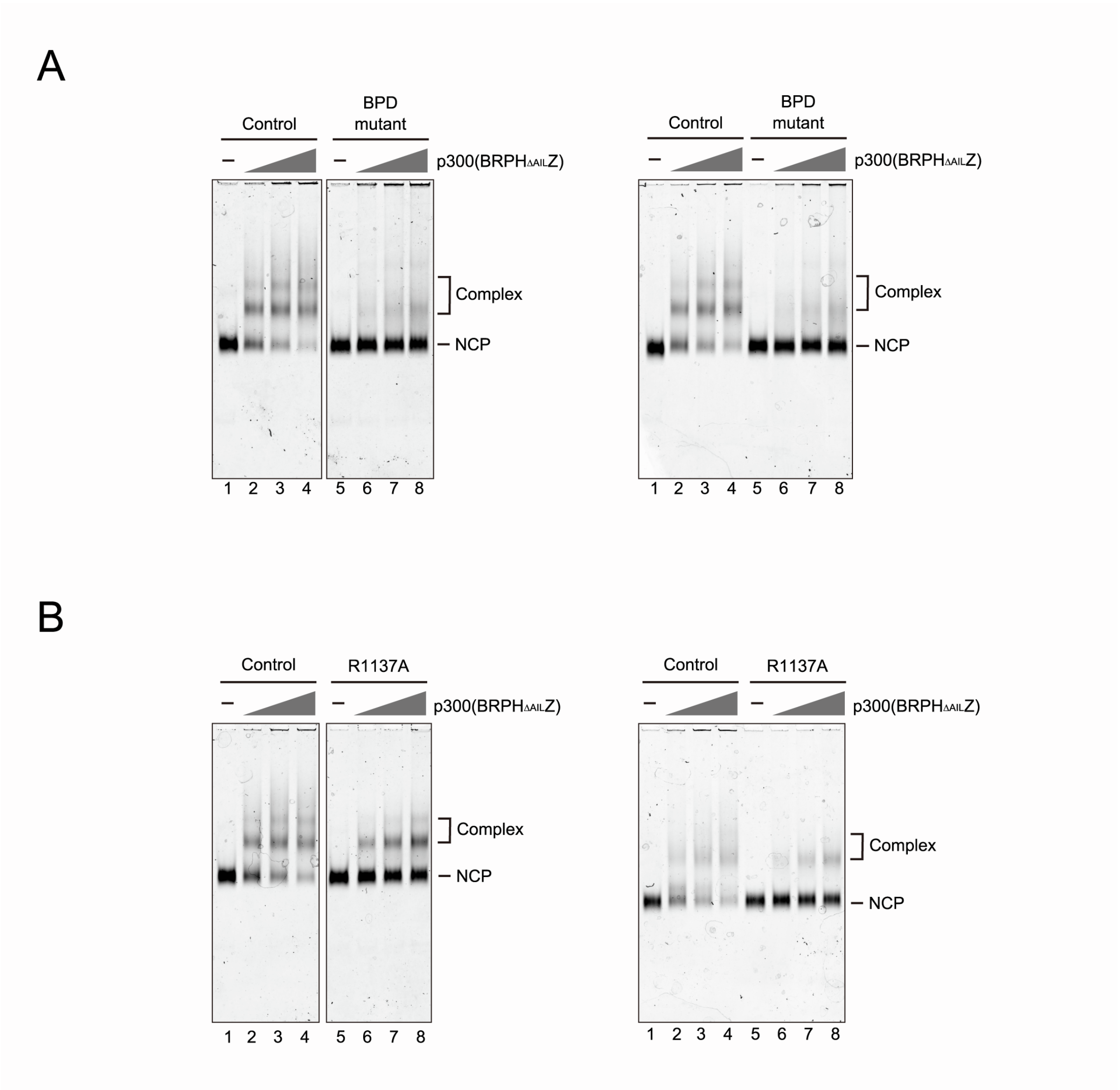

Figure S5. Replicated experiments related to Figure 3. (A) Electrophoretic mobility shift assay of the p300(BRPH$_{\Delta AIL}$Z) or p300(BRPH$_{\Delta AIL}$Z) BPD mutant (K1456A/K1459A/K1461A/R1462A) and the NCP. Complex formation was analyzed by non-denaturing 4% polyacrylamide gel electrophoresis with SYBR Gold staining. (B) Electrophoretic mobility shift assay of the p300(BRPH$_{\Delta AIL}$Z) or p300(BRPH$_{\Delta AIL}$Z) R1137A mutant and the NCP. Complex formation was analyzed by non-denaturing 4% polyacrylamide gel electrophoresis with SYBR Gold staining.
